# A high-sensitivity optical device for the early monitoring of plant pathogen attack via the *in vivo* detection of ROS bursts

**DOI:** 10.3389/fpls.2015.00096

**Published:** 2015-02-26

**Authors:** Lizhang Zeng, Jun Zhou, Bo Li, Da Xing

**Affiliations:** MOE Key Laboratory of Laser Life Science and Institute of Laser Life Science, College of Biophotonics, South China Normal UniversityGuangzhou, China

**Keywords:** optical devices, plant pathogens, plant pathogen attack, ROS burst, DCF

## Abstract

Biotic stressors, especially pathogenic microorganisms, are rather difficult to detect. In plants, one of the earliest cellular responses following pathogen infection is the production of reactive oxygen species (ROS). In this study, a novel optical device for the early monitoring of *Pseudomonas* attack was developed; this device measures the ROS level via oxidation-sensitive 2′, 7′-dichlorodihydrofluorescein diacetate (H_2DCFDA)-mediated_ fluorescence, which could provide early monitoring of attacks by a range of plant pathogen; ROS bursts were detected *in vivo* in *Arabidopsis thaliana* with higher sensitivity and accuracy than those of a commercial luminescence spectrophotometer. Additionally, the DCF fluorescence truly reflected early changes in the ROS level, as indicated by an evaluation of the H_2_O_2_ content and the tight association between the ROS and *Pseudomonas* concentration. Moreover, compared with traditional methods for detecting plant pathogen attacks based on physiological and biochemical measurements, our proposed technique also offers significant advantages, such as low cost, simplicity, convenient operation and quick turnaround. These results therefore suggest that the proposed optical device could be useful for the rapid monitoring of attacks by plant pathogen and yield results considerably earlier than the appearance of visual changes in plant morphology or growth.

## Introduction

As sessile creatures, plants are often challenged by a wide variety of environmental stress factors (Ma et al., 2009). Among these factors, biotic stressors, especially pathogenic microorganisms, are rather difficult to measure because their presence cannot be observed as directly as can abiotic factors, such as extreme temperature, ultraviolet radiation and osmotic stress; these abiotic factors can be measured directly or detected easily using a variety of means (Tenhaken et al., 1995). In fact, it has been demonstrated that plant-associated pathogens (which are called plant pathogens) have seriously threatened plant productivity and environmental safety, as well as the quality and diversity of food available (Chisholm et al., 2006; Jones and Dangl, 2006; Kamoun, 2006; Oerke, 2006; Tang et al., 2007). Therefore, an effective method for the early detection of plant pathogen attacks will play a vital role in restraining the proliferation of phytopathogenic microorganisms and reducing their impairments of plant growth and reproduction (Parry, 1990).

Conventional methods that are commonly employed to detect plant pathogen infection mainly rely on the identification of specific microbiological and biochemical components (Lindeberg et al., 2008; Velusamy et al., 2010). These methods can be divided into three categories: culture and colony-counting methods that involve pathogen counting (Löfström et al., 2004); nucleic acid-based methods, such as microarray analysis techniques and polymerase chain reaction techniques (Velusamy et al., 2010); and immunology-based methods that involve antigen-antibody interactions (Gehring et al., 2006). Although these methods can be sensitive and can provide both qualitative and quantitative information about the tested microorganisms, most of these techniques are severely restricted by the assay time and cost, as well as by their labor-intensive nature. Furthermore, initial enrichment is needed to detect pathogens, which typically occur in low numbers (Tang et al., 2007; Ma et al., 2009; Velusamy et al., 2010).

In plants, one of the most rapid and earliest cellular responses to plant pathogen infection is the so-called oxidative burst, which involves the production of reactive oxygen species (ROS), primarily superoxide (O^−^_2_) and hydrogen peroxide (H_2_O_2_), at the site of attempted invasion (Apostol et al., 1989; Land, 1990). Doke (1985) first reported the oxidative burst and demonstrated that potato tuber tissues generated O^−^_2_ that was rapidly transformed into H_2_O_2_ following inoculation with *Phytophthora infestans*. Subsequently, the ROS burst was documented in a number of plant-pathogen interactions (Grant et al., 2000; Bindschedler et al., 2001; Rojas et al., 2014), and enzymatic (Torres et al., 2002; Choi et al., 2007) and non-enzymatic sources (Yao and Greenberg, 2006) for ROS production during pathogen attack have been identified in plant cells (Allan and Fluhr, 1997; Apel and Hirt, 2004). In fact, a wide range of abiotic factors all appear to elicit ROS production in plant cells (Dong et al., 1991; Green and Fluhr, 1995; Inzé and Montagu, 1995; Zhang and Xing, 2008; Li et al., 2012; Baxter et al., 2014); however, these factors themselves can be identified directly by certain instruments. Detection of the ROS burst, therefore, may be a feasible strategy to monitor the early stages of phytopathogenic microorganism attack. To the best of our knowledge, however, there is currently no such pathogen detection system based on measuring ROS bursts *in vivo*.

Electron paramagnetic resonance is a classical method to measure the levels of ROS, and this method can measure and localize ROS *in vivo* (Halliwell and Whiteman, 2004). However, it is necessary to introduce spin trap molecules into the studied cells, which could potentially cause additional stress and affect the ROS levels (Shulaev and Oliver, 2006). Additionally, other methods have also been developed to measure superoxide and H_2_O_2_ in living tissues. The nitroblue tetrazolium (NBT) assay is used for the histochemical localization of superoxide (Flohe and Otting, 1984). Additionally, the measurement of H_2_O_2_ is generally based on the H_2_O_2_-dependent oxidation of a non-fluorescent substrate, such as the oxidation-sensitive 2′, 7′-dichlorodihydrofluorescein diacetate (H_2_DCFDA), which forms a fluorescent product that can be detected easily (Halliwell and Gutteridge, 1999; Steffens et al., 2013). It has been confirmed that H_2_DCFDA in 0.2% DMSO solution readily infiltrates epidermal cells of tobacco leaf tissue in the diacetate form (DCFH-DA), which is then hydrolyzed and trapped as non-fluorescent DCFH. Subsequent oxidation of DCFH by ROS yields the highly fluorescent dichlorofluorescein (DCF) (Allan and Fluhr, 1997).

Recently, optical devices have aroused considerable interest for the detection of foodborne pathogens and other plant physiology processes, such as seed germination and photosynthesis, due to their non-destructive nature and rapidity (Wang et al., 2007; Zhu et al., 2008; Liu et al., 2009; Velusamy et al., 2010). In this study, a novel optical device was developed for the early monitoring of plant pathogen attack; this device is based on direct and precise measurements of ROS bursts via H_2_DCFDA-mediated fluorescence *in vivo* in *Arabidopsis thaliana* (*A. thaliana*), allowing the early detection of plant pathogens.

## Materials and methods

### Materials

*A. thaliana* plants were grown in a plant growth chamber (Conviron, model E7/2, Winnipeg, Canada) with a 16 h light photoperiod (100 μmol photons m^−2^ s^−1^) and a relative humidity of 75/80% at 23/19°C (light/dark) for the whole experiment.

The bacterial strains *Pseudomonas syringae* pv. *tomato* DC3000 (*Pst* DC3000), *Pseudomonas syringae* pv. *phaseolicola* NPS3121 (*Psph* NPS3121) and *Pseudomonas syringae* pv. *maculicola* DG3 (*Psm* DG3) were provided by Professor Nan Yao (College of Life Science, Sun Yat-sen University, China). These plant pathogens were cultured according to the procedure described by Zhou et al. (2013).

### Pathogen infection

Three-week-old attached *A. thaliana* leaves were inoculated with a 1 ml suspension of *Pst* DC3000, *Psph* NPS3121 or *Psm* DG3 at various densities (**Figure 2**, Table 1) in 10 mM MgCl_2_ using a needleless syringe (Mishina and Zeier, 2007). All infection experiments were performed according to the procedure described by Matthews and Hull (2002) at room temperature in the presence of continuous light (100 μmol photons m^−2^ s^−1^). Control plants were treated with 10 mM MgCl_2_ solution. After infection for the required time interval, half (by number of detached leaves) of each group was used for ROS level measurements at 6 h after infection, and the remaining half was used to determine the concentration of *Pst* DC3000 based on the culture and colony-counting method after the necessary pathogen proliferation for 48 h.

**Table 1:** **Quantitative analysis of the suitability of the optical device for detecting the ROS production of *A. thaliana* leaves infected by plant pathogens**.

**Concentration (cfu ml^−**1**^)**	***Psph* NPS3121**		***Psm* DG3**	
	**Optical device**	**LS55**	**Optical device**	**LS55**
Control	48.2 ± 13.1	47.3 ± 18.7	46.6 ± 11.2	47.7 ± 16.8
10^1^	81.4 ± 8.3	55.2 ± 15.1	93.4 ± 8.3^*^	63.4 ± 18.3
10^2^	108.3 ± 9.2^*^	68.4 ± 12.5	208.2 ± 7.2^*^	100.2 ± 12.1^*^
10^3^	198.6 ± 8.1^*^	77.3 ± 18.1	338.1 ± 6.6^*^	238.2 ± 16.4^*^
10^4^	211.2 ± 9.2^*^	91.2 ± 23.1^*^	422.3 ± 5.2^*^	332.4 ± 15.5^*^
10^5^	220.1 ± 8.7^*^	198.3 ± 13.1^*^	501.1 ± 3.9^*^	451.6 ± 13.9^*^
10^6^	235.2 ± 6.4^*^	215.8 ± 10.4^*^	534.4 ± 11.7^*^	498.8 ± 16.2^*^
10^7^	313.5 ± 7.8^*^	233.5 ± 17.8^*^	632.1 ± 9.7^*^	532.3 ± 19.8^*^
10^8^	291.6 ± 6.1^*^	219.6 ± 10.1^*^	691.3 ± 8.2^*^	541.2 ± 18.9^*^

The mean ± SD of the DCF fluorescence intensity is shown for five independent A. thaliana leaves inoculated with Pseudomonas syringae pv. phaseolicola NPS3121 (Psph NPS3121) and Pseudomonas syringae pv. maculicola DG3 (Psm DG3) or 10 mM MgCl_2_ (Control). At 6 h after inoculation, sample leaves were detached and stained with 10 μ M H_2_DCFDA for 5 min in darkness, and the DCF fluorescence was assayed with the optical device and a commercial LS55 spectrophotometer as described in the Materials and Methods. There were a significant effect of DCF fluorescence obtained from optical device on the infected concentration with Psph NPS3121 (F_1, 8_ = 325.49, p < 0.01), DCF fluorescence obtained from LS55 on the infected concentration with Psph NPS3121 (F_1, 8_ = 81.684, p < 0.01), DCF fluorescence obtained from optical device on the infected concentration with Psm DG3 (F_1, 8_ = 2115.702, p < 0.01), DCF fluorescence obtained from LS55 on the infected concentration with Psm DG3 (F_1, 8_ = 437.054, p < 0.01).

* Numbers in columns followed by asterisks are significantly different from the MgCl_2_-treated control at P < 0.01 according to Duncan's multiple range test (DMRT).

### Pathogen growth assay

For the pathogen growth assay, leaves after 2 d of infection with unknown concentrations of *Pst* DC3000 were harvested. Disks of the leaves that were 0.37 cm in diameter and that originated from the infiltrated areas of five independent leaves were pounded into pieces in 10 mM MgCl_2_ in 1 ml Eppendorf tubes (Graham et al., 1990), then diluted for ten times and placed in Kings B medium containing the appropriate antibiotics for 3 d at 28°C, and the colony-forming units (cfu) were counted (Yao et al., 2004).

### Optical probe loading

H_2_DCFDA was obtained from Molecular Probes (Eugene, OR, USA) and dissolved in dimethyl sulfoxide (0.2% DMSO solution). The structure and spectral characteristics of H_2_DCFDA was shown in Supplementary Figure 1. After treatment with the pathogens for the required time, five leaves were incubated in 1.5 ml H_2_DCFDA (10 μM) in 0.2% DMSO solution for 5 min at room temperature in the dark and then rinsed with fresh distilled water to remove excess dye (Allan and Fluhr, 1997). The ROS production, indicated by the DCF fluorescence intensity, was measured using a custom-made optical device.

### Optical device system

#### Concept of operation

Two distinct operation modes, remote and local control, were designed for this system. The main instrument was operated using the front panel of the instrument in the local control mode and using a personal computer (PC) in the remote control mode. First, various parameters were set, including the excitation light intensity and time, the sample interval, the position of the sample and the experiment duration. Then, according to the defined parameters, the excitation light source irradiated the sample for the set excitation time. The intact leaves were placed in the sample chamber; the measurement was performed using a optical device that detected ultra-weak luminescence using the single-photon counting technique (Wang et al., 2007). The process of measuring the DCF fluorescence signal was divided into two steps: a background survey and a measurement of the mixed signal that included the DCF-oxidation fluorescence and the background. For the background, 10 μM H_2_DCFDA in 0.2% DMSO solution without leaves was used. The DCF fluorescence was obtained by subtracting the background from the mixed signal and was displayed directly on the front panel (local control mode) and on the PC display (remote control mode) relative to the DCF fluorescence.

#### System hardware design

**Figure 1:**
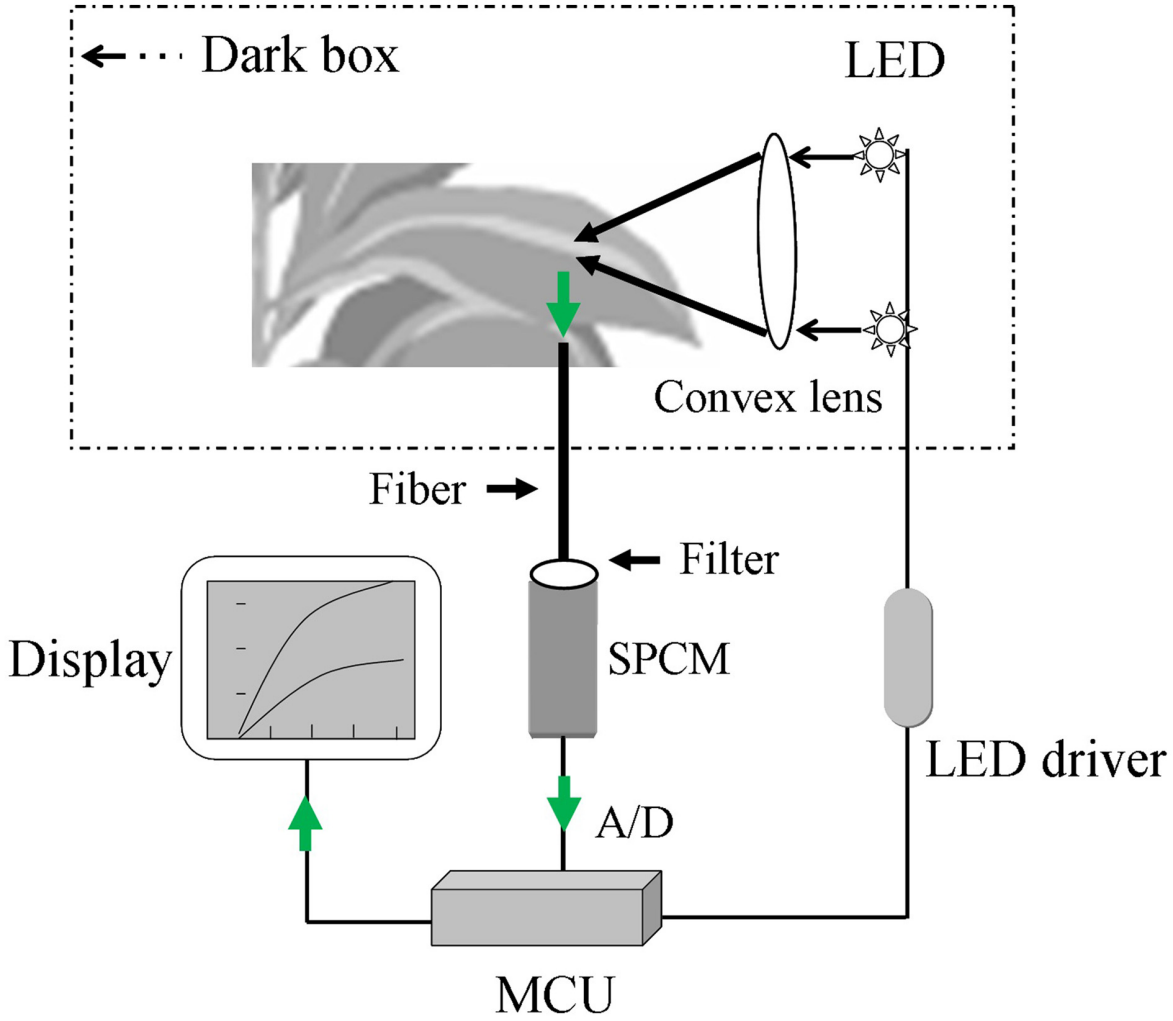
**A sketch of the custom-made sensor for detecting dichlorofluorescein (DCF) fluorescence**. The sensor is mainly composed of the following parts: sample chamber (dark box), excitation light source (light-emitting diode, LED), single photon counting module (SPCM), display system, and data acquisition and processing system (micro control unit, MCU). The green arrow represents the collection process for the DCF fluorescence signal.

A block diagram of the major hardware system for the optical device is shown in Figure 1. The system was mainly composed of the following hardware parts: dark sample chamber, excitation light source, ultra-high-sensitivity single photon counting module (SPCM; MP963, Perkin-Elmer, Wiesbaden, Germany), and a data acquisition and processing system. After infection and incubation with H_2_DCFDA, the samples were placed inside the sample chamber of the system and irradiated by a set of super bright light-emitting diodes (LEDs) (λ = 480 nm, full width at half maximum = 10 nm, single channel output luminous flux = 20 lm). The DCF fluorescence from the samples was collected using an optical fiber bundle and transmitted to the SPCM with a wavelength detection range of 185–850 nm. Because of the emission peak of DCF (Supplementary Figure 1), a 500–550 nm band-pass filter was used to avoid interference by the excitation light. The output signal, which had been amplified and discriminated by the SPCM, was collected and processed by a micro control unit (MCU; AT89c55) in the local control mode. The collected and processed signal could be stored in memory (AT29c020) before further data analysis using a PC. The total DCF fluorescence intensity between 500 and 550 nm was recorded to determine the relative ROS level.

#### Dark sample chamber

The dark chamber contained an upper chamber and a lower chamber for the adaxial and abaxial surfaces of the leaf, and these chambers were connected by a gemel. A protuberant ring and groove were located in the upper and lower chambers, respectively, and combined to form the dark cavity. To facilitate the placement of a live plant leaf in the sample chamber, an air hole was placed in a black hermetic foam cushion, which was used to exert non-destructive tension on the leaf stalk while shielding the sample from outside light. The dark sample chamber was locked and opened using a lock and spring.

#### LED driver

To stabilize the excitation light intensity produced by the LED, a low-noise constant-current source was employed at the output level; this source was able to stabilize the current with only a small ripple coefficient. A high-power field effect transistor was used to drive the LED light source. An automatic current control feedback technique was used to stabilize the positive drive current and provide continuous regulation in the range of 0–100 mA with a control precision of approximately 0.1 mA.

### LS55 spectrophotometer

To test the accuracy of this system at quantifying the ROS level, the DCF fluorescence of the sample was also determined using a commercial luminescence spectrophotometer (LS55, Perkin-Elmer, UK) according to a previously described procedure (Allan and Fluhr, 1997; Zhang and Xing, 2008) using an excitation wavelength of 480 nm and emission wavelengths between 500 and 550 nm (excitation and emission slit widths of 5 nm). The fluorescence intensity was obtained by performing an integration between 500 and 550 nm to determine the relative ROS level.

### Determination of H_2_O_2_ content

To test the accuracy of using H_2_DCFDA to quantify the ROS level, the H_2_O_2_ content was also examined using a hydrogen peroxide assay kit (Beyotime Biotech, China). H_2_O_2_oxidizes Fe^2+^ to Fe^3+^, and Fe^3+^ can react with xylenol orange in a colorimetric reaction. Briefly, a 50 mg sample of infected leaves was ground in 500 μl lysis buffer and then centrifuged at 12,000 g for 5 min; the resulting supernatant was retained for the subsequent detection. Next, 50 μl supernatant and 100 μl test solution were placed at room temperature for 30 min, and the fluorescence was measured directly using a commercial luminescence spectrophotometer (LS55, Perkin-Elmer, UK) at a wavelength of 560 nm. The concentration of H_2_O_2_ was calculated from a standard concentration curve (Dai et al., 2010), (Supplementary Figure 2).

### Statistical analysis

Data were subjected to mean separation by Duncan's multiple range tests (DMRTs; *p* < 0.05) and significance by analysis of variance (ANOVA) using SPSS 12.0 software for Windows (Nyaboga et al., 2014). To stabilize the variance and fulfill the normality assumption of ANOVAs, log10 transformations were applied. Correlations between DCF fluorescence from LS55 and DCF fluorescence from optical device were tested by means of the Pearson correlation coefficient (Liu et al., 2010).

## Results

### Concentration-dependent effects of plant pathogens on ROS production

**Figure 2:**
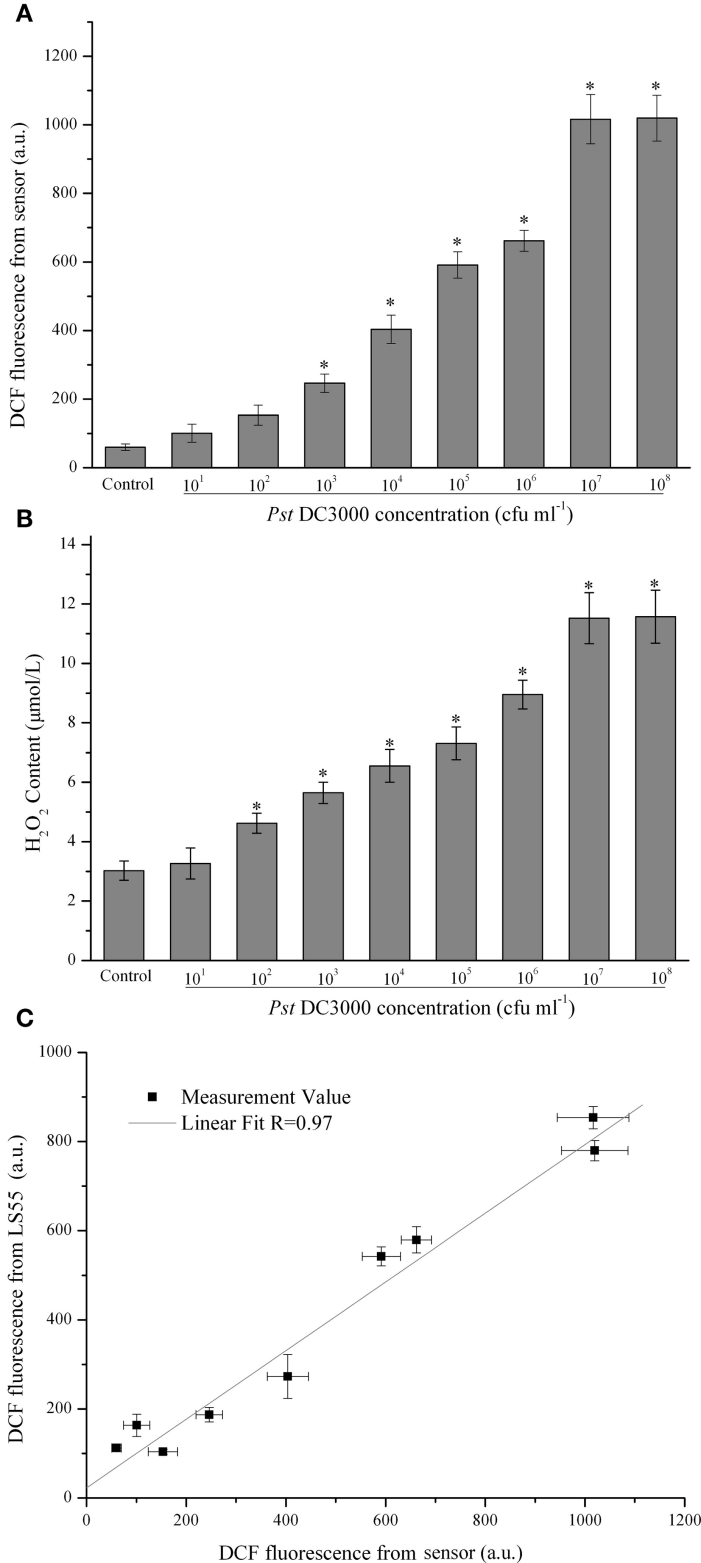
**Quantitative analysis of concentration-dependent changes in the dichlorofluorescein (DCF) fluorescence intensity of *Arabidopsis thaliana (A. thaliana)* leaves infected with *Pseudomonas syringae* pv*.tomato* DC3000 (*Pst* DC3000)**. The DCF fluorescence was determined by the optical device with difference concentration of Pst DC3000 infection **(A)**. The mean ± SD of the fluorescence intensity is shown for five independent *A. thaliana* leaves inoculated with *Pst* DC3000 or 10 mM MgCl_2_ (Control). There were a significant effect of DCF fluorescence obtained from optical device on the infected concentration with *Pst* DC3000 (*F*_1, 8_ = 228.179, *p* < 0.01). The H_2_O_2_ content was determined according to the standard procedure of a hydrogen peroxide assay kit **(B)**. There were a significant effect of ROS content obtained from optical device on the infected concentration with *Pst* DC3000 (*F*_1, 8_ = 97.519, *p* < 0.01). The asterisks indicate significant differences (*P* < 0.01) compared with the MgCl_2_-treated control according to Duncan's multiple range test (DMRT). The DCF fluorescence was also assayed using a LS55 spectrophotometer as described in the Materials and Methods, and the relationship between the DCF fluorescence detected by the LS55 spectrophotometer and the DCF fluorescence detected by the developed sensor was analyzed **(C)**. A.U., arbitrary unit; cfu, colony-forming units; df, degree of freedom.

In this study it was demonstrated that as the *Pst* DC3000 concentration increased, the ROS level initially increased, reaching a maximum value at 10^7^ cfu (colony-forming units) ml^−1^ and then decreasing slightly at 10^8^ cfu ml^−1^ (Figure 2A). When the concentration of *Pst* DC3000 increased to 10^3^ cfu ml^−1^, a significant increase in the DCF fluorescence intensity was observed compared with that for the MgCl_2_ treatment (Control, Figure 2A). A similar result was obtained when the H_2_O_2_ content was measured (Figure 2B). It should be emphasized that no disease lesion was visible even after 18 h of infection with 10^6^ cfu ml^−1^ of the pathogen (data not shown). Interestingly, the changes in the DCF fluorescence level in *A. thaliana* leaves infected with various amounts of *Pst* DC3000 detected by the commercial LS55 spectrophotometer showed a trend similar to that detected by the developed sensor (data not shown). Statistical analyses revealed a good positive correlation between these two types of data, which were detected by two distinct systems (*R* = 0.97, Figure 2C), indicating that the proposed optical device could detect the ROS level with high accuracy.

### The early monitoring of plant pathogen attack

**Figure 3:**
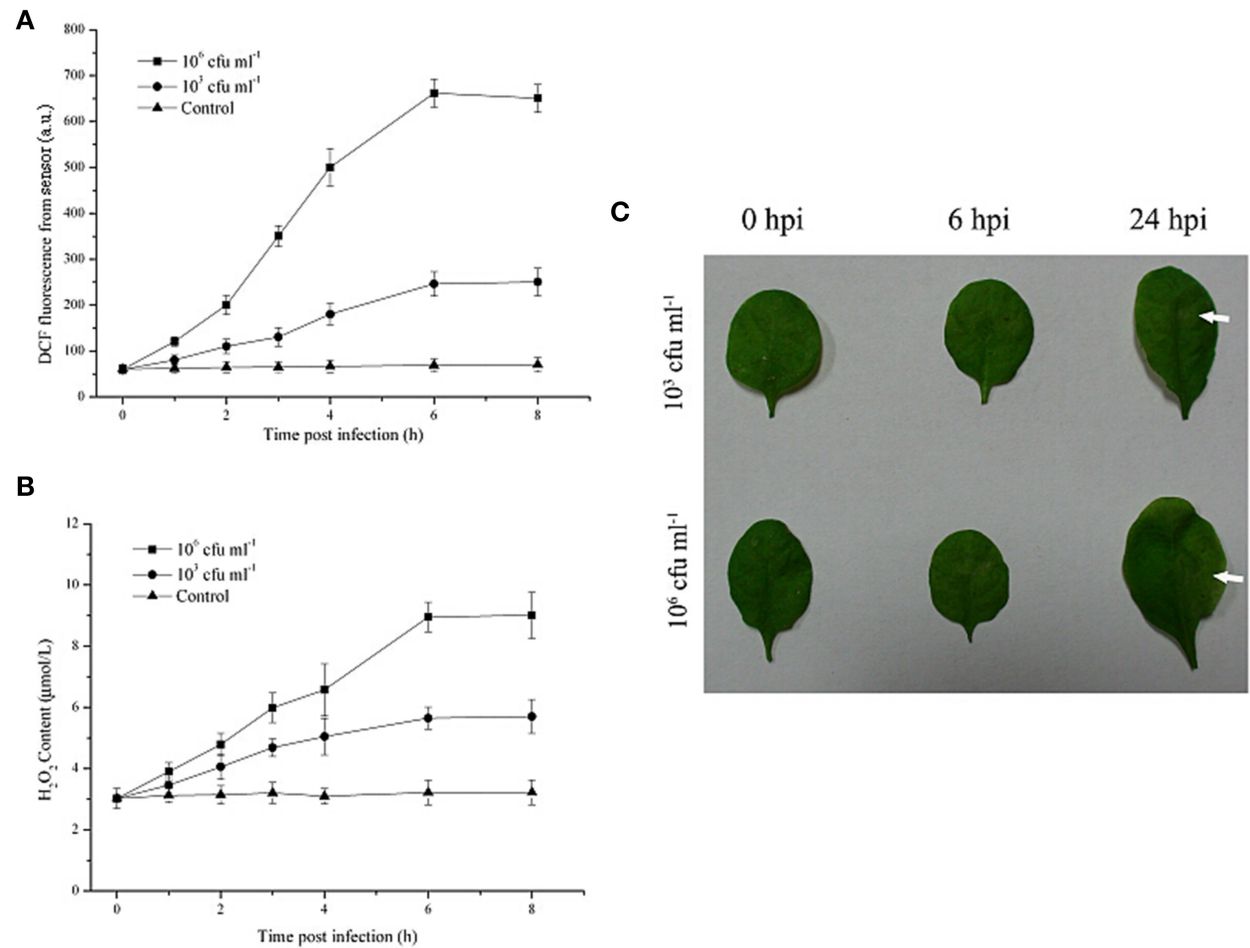
**The early monitoring of *Pseudomonas syringae* pv. *tomato* DC3000 (*Pst* DC3000) infection in *A. thaliana* leaves by the optical sensor through detection of the ROS production dynamics**. The DCF fluorescence was determined by the optical device at 2, 4, 6, 8 hours post infection with 10^6^ cfu ml^−1^ or 10^3^ cfu ml^−1^ Pst DC3000 **(A)**. The mean ± SD of the fluorescence intensity is shown for five independent *A. thaliana* leaves inoculated with 10^3^ cfu ml^−1^ or 10^6^ cfu ml^−1^ of the pathogen or 10 mM MgCl_2_ (Control). The H_2_O_2_ content was determined according to the standard procedure of a hydrogen peroxide assay kit **(B)**. **(C)** Macroscopic disease development (*white arrows*) of *A. thaliana* leaves infected by *Pst* DC3000. Independent experiments were repeated five times with similar results. Bars = 3 mm. A.U., arbitrary unit; cfu, colony-forming units; hpi, hour post inoculation.

As shown in Figure 3, in the presence of *Pst* DC3000 at 10^6^ cfu ml^−1^, a robust and long ROS burst could be detected by this optical system; the increased DCF fluorescence in the leaves reached a peak at 6 h after inoculation (Figure 3A). Moreover, upon infection with the limiting concentration (10^3^ cfu ml^−1^), a pronounced and long ROS burst could also be detected by the optical device (Figure 3A). The increased DCF fluorescence in the leaves reached a peak at the same infection time and remained detectable until 8 h after inoculation, although at any given time point, the DCF fluorescence level in leaves infected with 10^3^ cfu ml^−1^ of the pathogen was lower than that in leaves infected with 10^6^ cfu ml^−1^ of *Pst* DC3000 (Figure 3A). In contrast, the leaves inoculated with 10 mM MgCl_2_ (Control) exhibited no apparent increase in ROS production throughout the assessment period (Figure 3A). The H_2_O_2_ content showed a similar pattern. Additionally, it should be noted that no visible disease lesion could be found until 24 h post inoculation (hpi, Figure 3B). Taken together, these results demonstrated that the developed optical device could provide early monitoring of the *Pst* DC3000 infection in real time during a time period that was considerably earlier than the onset of any visual effects on plant morphology or growth.

### Practical application of plant pathogen infection monitoring

**Figure 4:**
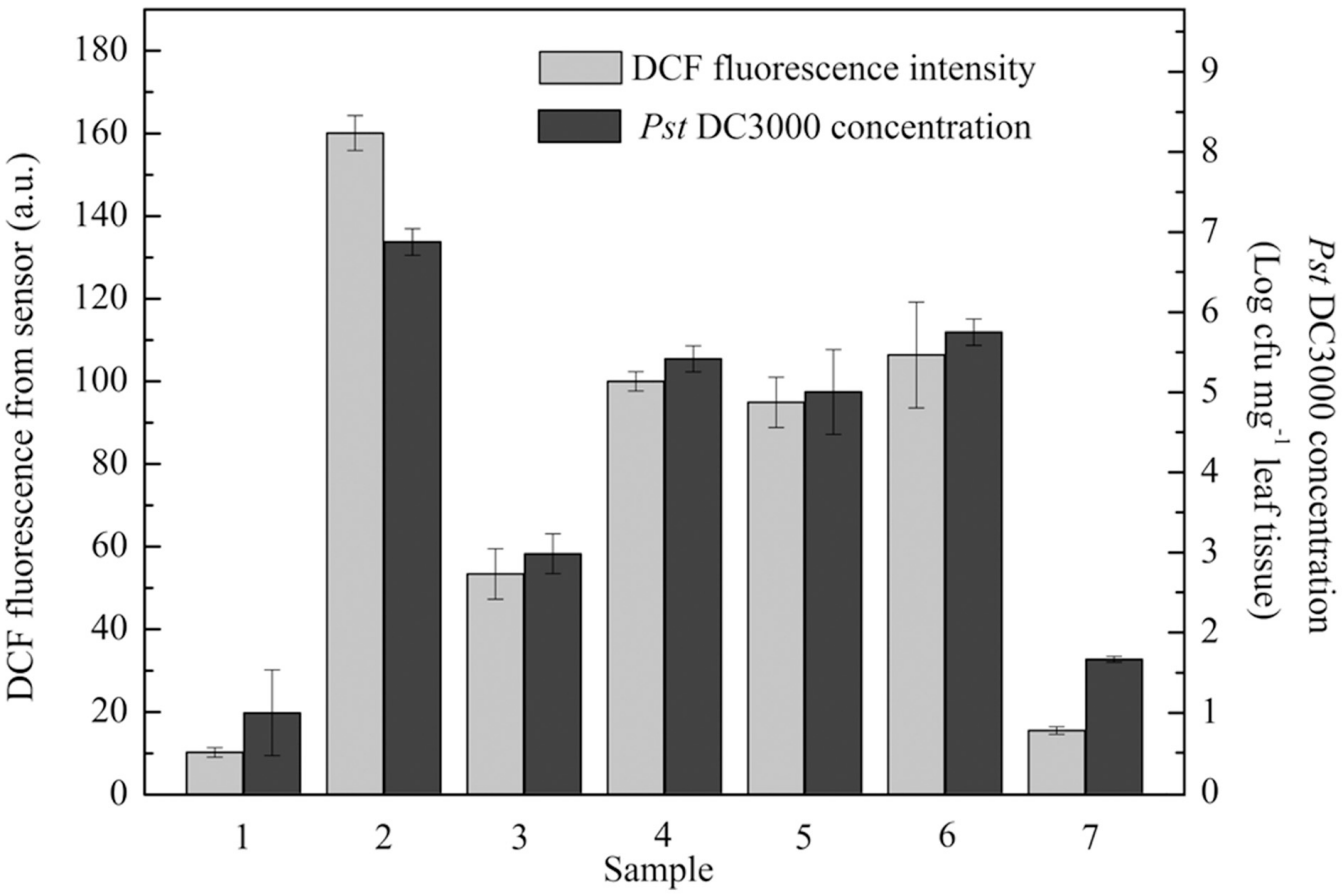
**A series of unknown concentrations of *Pseudomonas syringae* pv*. tomato* DC3000 (*Pst* DC3000) were used to infect *A. thaliana* leaves**. Dichlorofluorescein (DCF) fluorescence was measured at 6 hpi. The concentration of *Pst* DC3000 was measured by conventional methods based on the culture and colony-counting method following 48 h of proliferation as described in the Materials and Methods. The mean ± SD of the fluorescence intensity represents the amounts of pathogen in five independent *A. thaliana* leaves. A.U., arbitrary unit; cfu, colony-forming units; hpi, hour post inoculation.

A practical application of the proposed optical device for monitoring plant pathogen attack under stochastic situations was also tested. Unknown concentrations of *Pst* DC3000 were used to stochastically infect seven groups of *A. thaliana* leaves (Groups 1, 2, 3, 4, 5, 6, and 7). As shown in Figure 4, the level of ROS production in each group detected by the proposed optical device at 6 hpi clearly reflected the amounts of *Pst* DC3000, while the conventional methods based on the culture and colony-counting method required 2 d to determine the amounts of *Pst* DC3000.

### Feasibility of detecting other plant pathogen infections

The suitability of the proposed system for monitoring attacks by other plant pathogen was also tested. *A. thaliana* leaves were inoculated with two other plant pathogens (*Psm* DG3 and *Psph* NPS3121; at concentrations ranging from 10^1^ cfu ml^−1^ to 10^8^ cfu ml^−1^), and the ROS production was measured at 6 h using the proposed system and using a commercial LS55 spectrophotometer. The result indicated that in response to each of the two plant pathogens, the proposed system detected similar trends in the ROS level in response to the two pathogens (Table 1). Importantly, when the concentrations of *Psph* NPS3121 and *Psm* DG3 were 10^2^ and 10^1^ cfu ml^−1^, respectively, the ROS levels measured by the developed optical device were significantly different from those of the control at *P* < 0.01 (Table 1); thus, the proposed system provided greater sensitivity than that of the commercial LS55 spectrophotometer when detecting ROS, indicating that the developed optical device could be used to monitor attack by a variety of plant pathogens with high sensitivity.

**Figure 5:**
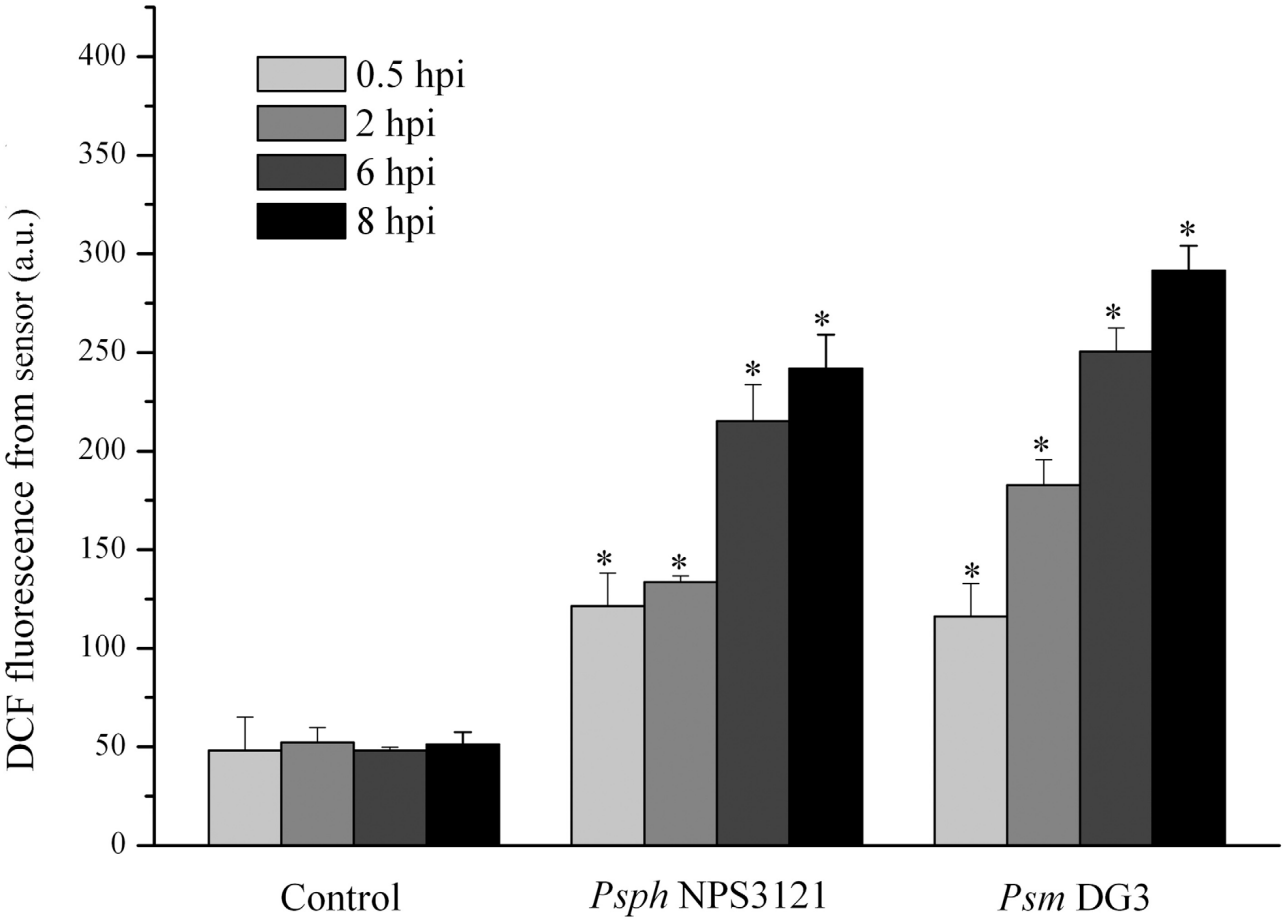
**Quantitative analysis of time-dependent changes in the dichlorofluorescein (DCF) fluorescence in *A. thaliana* leaves infected by 10^3^ cfu ml^−1^*Pseudomonas syringae* pv. *phaseolicola* NPS3121 (*Psph* NPS3121) (*Psph* NPS3121) and 10^2^ cfu ml^−1^*Pseudomonas syringae* pv. *maculicola* DG3 (*Psm* DG3) in 10 mM MgCl_2_**. The mean ± SD of the fluorescence intensity is shown for five independent *A. thaliana* leaves inoculated with various pathogens or 10 mM MgCl_2_ (Control). There were a significant effect of DCF fluorescence obtained from optical device on the type of infected plant pathogen at 0.5 hpi (*F*_1, 2_ = 108.413, *p* < 0.01), that at 2 hpi (*F*_1, 2_ = 979.962, *p* < 0.01), that at 6 hpi (*F*_1, 2_ = 1125.01, *p* < 0.01), that at 8 hpi (*F*_1, 2_ = 263.57, *p* < 0.01). Asterisks (*) indicate significant differences from control at the indicated time points at *P* < 0.01 according to Duncan's multiple range test (DMRT). A.U., arbitrary unit; cfu, colony-forming units; df, degree of freedom.

Next, the limiting concentration of two plant pathogens (*Psm* DG3 at 10^2^ cfu ml^−1^ and *Psph* NPS3121 at 10^3^ cfu ml^−1^) was also used to infect *A. thaliana* leaves, and the dynamics of DCF fluorescence were measured. The results demonstrated that all pathogens used here could result in a robust ROS burst compared with the response to MgCl_2_ treatment. Moreover, the ROS level in the leaves infected with both pathogens was significantly higher than that in the control samples at all indicated time points (Figure 5). It should be noted that, under infection by these pathogens, no visible disease lesion could be found until 24 hpi (data not shown). Based on these results, we can therefore conclude that this proposed approach can be successfully used to detect several pathogen attacks considerably earlier than the observation of disease lesions.

## Discussion

The feasibility of the developed optical device has been validated in *A. thaliana*. The current investigation has demonstrated that the proposed system could provide early monitoring of the attack of plant pathogens *in vivo* considerably earlier than the onset of any visual effects on plant morphology or growth, displaying wide application prospects.

In this study, a novel plant pathogen attack sensor was developed based on the quantitative measurement of ROS bursts *in vivo*. The novel features of the optical device described here include the following. (1) The optical device was developed based on a new idea: measuring plant pathogen attacks using ROS bursts, which is one of the most rapid and earliest cellular responses following plant pathogen infection. Therefore, the optical device could be effectively used to monitor attacks by plant pathogen microorganisms considerably earlier than the onset of any visually observable effects of the disease lesion on the leaf morphology or plant growth. (2) Compared with conventional methods, our proposed method for detecting plant pathogen infection is less time-consuming, much easier and more convenient and might therefore be widely used in pest management. Performing our new method only involves placing pathogen-infected leaves into H_2_DCFDA solution for several minutes of incubation in the dark and fluorescence collection; the procedure only required approximately 10 min. While the conventional methods commonly employed to detect plant pathogen infection, such as microarray analysis, polymerase chain reactions and immunology techniques, require long sample preparation times (usually over several hours) and complicated extraction processes. However, according to the detection of DCF-based ROS levels, the method cannot distinguish between plant pathogen species. (3) The optical device has higher sensitivity and accuracy at detecting plant pathogen infection via measuring the ROS level than those of commercial instruments; moreover, the optical device could be used to detect ROS bursts caused by plant pathogen infection *in vivo*, whereas commercial instruments, such as the LS55 spectrophotometer, are only capable of measuring ROS bursts in trimmed segments of detached leaves. (4) The only reaction reagent in the proposed method is H_2_DCFDA; theoretically, the cost of each test is very low. The new method has the merits of low cost, simple and convenient operation, and remote control, making it a strong competitor for early, high-sensitivity examinations and extended inspections of plant pathogen infection, as well as for wide application in precision agriculture. In a previous study, Choi et al. (2012) indicated that DCF-based ROS sensors have significant limitations and that DCF-based H_2_O_2_ probes are susceptible to photobleaching and photooxidation. To test the accuracy of H_2_DCFDA for quantifying the ROS level using this system, the H_2_O_2_ content was also examined by a hydrogen peroxide assay kit. The consistent results confirmed the accuracy of this probe at detecting infections by plant pathogens in this system. (5) The optical device is portable because it relies on SPCM and MCU techniques. (6) The optical device provided a localized assay *in vivo* using a hermetic dark sample chamber and a battery power supply. The proposed strategy is simple and does not require skilled workers to perform the tests, and it can provide rapid and early reliable data on the tested microorganisms. With further development, the system should be able to provide *in situ* detection of pathogenic microorganisms and would have potential applications in the preliminary evaluation of pathogen attack before further complicated, cost- and time-consuming biochemical analyses.

### Conflict of interest statement

The authors declare that the research was conducted in the absence of any commercial or financial relationships that could be construed as a potential conflict of interest.

## Supplementary material

Supplementary Figure 1 **Structure and spectral characteristics of H_**2**_DCFDA**. **(A)** Molecular structure of H_2_DCFDA. **(B)** The absorption spectrum of 10 μ M H_2_DCFDA in 0.2% DMSO solution shows a maximum absorption peak at 502 nm. In all experiments, however, we used 480 nm as the excitation light to avoid the effect of the excitation light on the fluorescence emission. **(C)** Fluorescence emission spectra of 10 μ M H_2_DCFDA in 0.2% DMSO solution with excitation at 480 nm. **(D)** Fluorescence emission spectra of *A. thaliana* leave after incubation with 10 μ M H_2_DCFDA in 0.2% DMSO solution. The samples were excited at 480 nm, and the spectra show a 6 nm redshift when compared with **(C)**. All spectra were obtained using an LS55 spectrophotometer in the presence of 10 μ M H_2_O_2_ applied exogenously to oxidize the H_2_DCFDA.

Supplementary Figure 2 **Standard concentration curve of H_2_O_2_**.

## References

[B1] Allan A. C. Fluhr R. (1997). Two distinct sources of elicited reactive oxygen species in tobacco epidermal cells. Plant Cell 9, 1559–1572. 10.1105/tpc.9.9.1559 12237396 PMC157033

[B2] Apel K. Hirt H. (2004). Reactive oxygen species: metabolism, oxidative stress, and signal transduction. Annu. Rev. Plant Biol. 55, 373–399. 10.1146/annurev.arplant.55.031903.141701 15377225

[B3] Apostol I. Heinstein P. F. Low P. S. (1989). Rapid stimulation of an oxidative burst during elicitation of cultured plant cells role in defense and signal transduction. Plant Physiol. 90, 109–116. 10.1104/pp.90.1.109 16666719 PMC1061684

[B4] Baxter A. Mittler R. Suzuki N. (2014). ROS as key players in plant stress signalling. J. Exp. Bot. 65, 1229–1240. 10.1093/jxb/ert375 24253197

[B5] Bindschedler L. V. Minibayeva F. Gardner S. L. Gerrish C. Davies D. R. Bolwell G. P. (2001). Early signalling events in the apoplastic oxidative burst in suspension cultured French bean cells involve cAMP and Ca^2+^. New Phytol. 151, 185–194 10.1046/j.1469-8137.2001.00170.x 33873377

[B6] Chisholm S. T. Coaker G. Day B. Staskawicz B. J. (2006). Host-microbe interactions: shaping the evolution of the plant immune response. Cell 124, 803–814. 10.1016/j.cell.2006.02.008 16497589

[B7] Choi H. W. Kim Y. J. Lee S. C. Hong J. K. Hwang B. K. (2007). Hydrogen peroxide generation by the pepper extracellular peroxidase CaPO_2_ activates local and systemic cell death and defense response to bacterial pathogens. Plant Physiol. 145, 890–904 10.1104/pp.107.103325 17905862 PMC2048806

[B8] Choi W. G. Swanson S. J. Gilroy S. (2012). High-resolution imaging of Ca^2+^, redox status, ROS and pH using GFP biosensors. Plant J. 70, 118–128. 10.1111/j.1365-313X.2012.04917.x 22449047

[B9] Dai X. Sun Y. Gao Z. Jiang Z. (2010). Copper enhances amyloid-β peptide neurotoxicity and non β-aggregation: a series of experiments conducted upon copper-bound and copper-free amyloid-β peptide. J. Mol. Neurosci. 41, 66–73. 10.1007/s12031-009-9282-8 19685013

[B10] Doke N. (1985). NADPH-dependent O2-generation in membrane fractions isolated from wounded potato tubers inoculated with Phytophthora infestans. Physiol. Plant. Pathol. 27, 311–322 10.1016/0048-4059(85)90044-X

[B11] Dong X. Mindrinos M. Davis K. R. Ausubel F. M. (1991). Induction of Arabidopsis defense genes by virulent and avirulent *Pseudomonas syringae* strains and by a cloned avirulence gene. Plant Cell 3, 61–72. 10.1105/tpc.3.1.61 1824335 PMC159979

[B12] Flohe L. Otting F. (1984). Superoxide dismutase assays. Methods Enzymol. 105, 93–104. 10.1016/S0076-6879(84)05013-8 6328209

[B13] Gehring A. G. Irwin P. L. Reed S. A. Tu S. I. (2006). Enzyme-linked immunomagnetic chemiluminescence incorporating anti-H7 and anti-O157 antibodies for the detection of Escherichia coli O157:H7. J. Rapid Meth. Aut. Mic. 14, 349–361 10.1111/j.1745-4581.2006.00059.x

[B14] Graham J. H. Hartung J. S. Stall R. E. Chase A. R. (1990). Pathological, restriction fragment length polymorphism, and fatty acid profile relationships between *Xanthomonas campestris* from citrus and noncitrus hosts. Phytopathol. 80, 829–836 10.1094/Phyto-80-829

[B15] Grant M. Brown I. Adams S. Knight M. Ainslie A. Mansfield J. (2000). The RPM1 plant disease resistance gene facilitates a rapid and sustained increase in cytosolic calcium that is necessary for the oxidative burst and hypersensitive cell death. Plant J. 23, 441–450. 10.1046/j.1365-313x.2000.00804.x 10972870

[B16] Green R. Fluhr R. (1995). UV-B-induced PR-1 accumulation is mediated by active oxygen species. Plant Cell 7, 203–212. 10.1105/tpc.7.2.203 12242373 PMC160776

[B16a] Halliwell B. Gutteridge J. M. C. (1999). Free Radicals in Biology and Medicine, 3rd Edn Oxford, UK: Oxford University Press.

[B17] Halliwell B. Whiteman M. (2004). Measuring reactive species and oxidative damage *in vivo* and in cell culture: how should you do it and what do the results mean? Br. J. Pharmacol. 142, 231–255. 10.1038/sj.bjp.0705776 15155533 PMC1574951

[B18] Inzé D. Montagu M. V. (1995). Oxidative stress in plants. Curr. Opin. Biotechnol. 6, 153–158 10.1016/0958-1669(95)80024-7

[B19] Jones J. D. Dangl J. L. (2006). The plant immune system. Nature 444, 323–329. 10.1038/nature05286 17108957

[B20] Kamoun S. (2006). A catalogue of the effector secretome of plant pathogenic oomycetes. Annu. Rev. Phytopathol. 44, 41–60. 10.1146/annurev.phyto.44.070505.143436 16448329

[B21] Land E. T. (1990). Free radicals in biology and medicine. Int. J. Radiat. Biol. 58, 725–725. 10.1080/095530090145520716297065

[B22] Li Z. Xing F. Xing D. (2012). Characterization of target site of aluminum phytotoxicity in photosynthetic electron transport by fluorescence techniques in tobacco leaves. Plant Cell Physiol. 53, 1295–1309 10.1093/pcp/pcs076 22611177

[B23] Lindeberg M. Myers C. R. Collmer A. Schneider D. J. (2008). Roadmap to new virulence determinants in Pseudomonas syringae: insights from comparative genomics and genome organization. Mol. Plant Microbe Interact. 21, 685–700. 10.1094/MPMI-21-6-0685 18624633

[B24] Liu F. Tang Y. Du R. Yang H. Wu Q. Qiu R. (2010). Root foraging for zinc and cadmium requirement in the Zn/Cd hyperaccumulator plant Sedum alfredii. Plant Soil 327, 365–375 10.1007/s11104-009-0060-8

[B25] Liu X. Gao C. Xing D. (2009). A non-invasive and rapid seed vigor biosensor based on quantitative measurement of superoxide generated by aleurone cell in intact seeds. Biosens. Bioelectron. 24, 1537–1542. 10.1016/j.bios.2008.06.040 19338074

[B26] Löfström C. Knutsson R. Axelsson C. E. Rådström P. (2004). Rapid and specific detection of Salmonella spp. In animal feed samples by PCR after culture enrichment. Appl. Environ. Microbiol. 70, 69–75. 10.1128/AEM.70.1.69-75.2004 14711627 PMC321250

[B27] Ma W. Qi Z. Smigel A. Walker R. K. Verma R. Berkowitz G. A. (2009). Ca^2+^, cAMP, and transduction of non-self perception during plant immune responses. Proc. Natl. Acad. Sci. U.S.A. 106, 20995–21000. 10.1073/pnas.0905831106 19933332 PMC2780315

[B28] Matthews R. E. F. Hull R. (2002). Matthews' Plant Virology. San Diego: Elsevier.

[B29] Mishina T. E. Zeier J. (2007). Pathogen-associated molecular pattern recognition rather than development of tissue necrosis contributes to bacterial induction of systemic acquired resistance in Arabidopsis. Plant J. 50, 500–513. 10.1111/j.1365-313X.2007.03067.x 17419843

[B30] Nyaboga E. Tripathi J. N. Manoharan R. Tripathi L. (2014). Agrobacterium-mediated genetic transformation of yam (Dioscorea rotundata): an important tool for functional study of genes and crop improvement. Front. Plant Sci. 5:463. 10.3389/fpls.2014.00463 25309562 PMC4164099

[B31] Oerke E. C. (2006). Crop losses to pests. J Agric Sci. 144, 31–43 10.1017/S0021859605005708

[B32] Parry D. W. (1990). Plant Pathology in Agriculture. New York, NY: Cambridge University Press.

[B33] Rojas C. M. Senthil-Kumar M. Tzin V. Mysore K. S. (2014). Regulation of primary plant metabolism during plant-pathogen interactions and its contribution to plant defense. Front. Plant Sci. 5:17. 10.3389/fpls.2014.00017 24575102 PMC3919437

[B34] Shulaev V. Oliver D. J. (2006). Metabolic and proteomic markers for oxidative stress. New tools for reactive oxygen species research. Plant Physiol. 141, 367–372. 10.1104/pp.106.077925 16760489 PMC1475455

[B35] Steffens B. Steffen-Heins A. Sauter M. (2013). Reactive oxygen species mediate growth and death in submerged plants. Front. Plant Sci. 4:179. 10.3389/fpls.2013.00179 23761805 PMC3671184

[B36] Tang Y. B. Xing D. Zhu D. B. Liu J. F. (2007). An improved electrochemiluminescence polymerase chain reaction method for highly sensitive detection of plant viruses. Anal. Chim. Acta. 582, 275–280. 10.1016/j.aca.2006.09.021 17386503

[B37] Tenhaken R. Levine A. Brisson L. F. Dixon R. A. Lamb C. (1995). Function of the oxidative burst in hypersensitive disease resistance. Proc. Natl. Acad. Sci. U.S.A. 92, 4158–4163. 10.1073/pnas.92.10.4158 11607542 PMC41903

[B38] Torres M. A. Dangl J. L. Jones J. D. (2002). Arabidopsis gp91phox homologues AtrbohD and AtrbohF are required for accumulation of reactive oxygen intermediates in the plant defense response. Proc. Natl. Acad. Sci. U.S.A. 99, 517–522. 10.1073/pnas.012452499 11756663 PMC117592

[B39] Velusamy V. Arshak K. Korostynska O. Oliwa K. Adley C. (2010). An overview of foodborne pathogen detection: in the perspective of biosensors. Biotechnol. Adv. 28, 232–254. 10.1016/j.biotechadv.2009.12.004 20006978

[B40] Wang J. Xing D. Zhang L. Jia L. (2007). A new principle photosynthesis capacity biosensor based on quantitative measurement of delayed fluorescence *in vivo*. Biosens. Bioelectron. 22, 2861–2868. 10.1016/j.bios.2006.12.007 17229566

[B41] Yao N. Eisfelder B. J. Marvin J. Greenberg J. T. (2004). The mitochondrion–an organelle commonly involved in programmed cell death in *Arabidopsis thaliana*. Plant J. 40, 596–610. 10.1111/j.1365-313X.2004.02239.x 15500474

[B42] Yao N. Greenberg J. T. (2006). Arabidopsis ACCELERATED CELL DEATH2 modulates programmed cell death. Plant Cell 18, 397–411. 10.1105/tpc.105.036251 16387834 PMC1356547

[B43] Zhang L. Xing D. (2008). Methyl jasmonate induces production of reactive oxygen species and alterations in mitochondrial dynamics that precede photosynthetic dysfunction and subsequent cell death. Plant Cell Physiol. 49, 1092–1111. 10.1093/pcp/pcn086 18535010

[B44] Zhou J. Sun A. Xing D. (2013). Modulation of cellular redox status by thiamine-activated NADPH oxidase confers Arabidopsis resistance to *Sclerotinia sclerotiorum*. J. Exp. Bot. 64, 3261–3272. 10.1093/jxb/ert166 23814275

[B45] Zhu D. Tang Y. Xing D. Chen W. R. (2008). PCR-free quantitative detection of genetically modified organism from raw materials. An electrochemiluminescence-based bio bar code method. Anal. Chem. 80, 3566–3571. 10.1021/ac0713306 18386909 PMC5978678

