# Supplementary figures and images for: A high-sensitivity optical device for the early monitoring of plant pathogen attack via the in vivo detection of ROS bursts
Source: Front Plant Sci. 2015 Feb 26;6:96. doi: 10.3389/fpls.2015.00096 (PMC4341508; doi:10.3389/fpls.2015.00096)

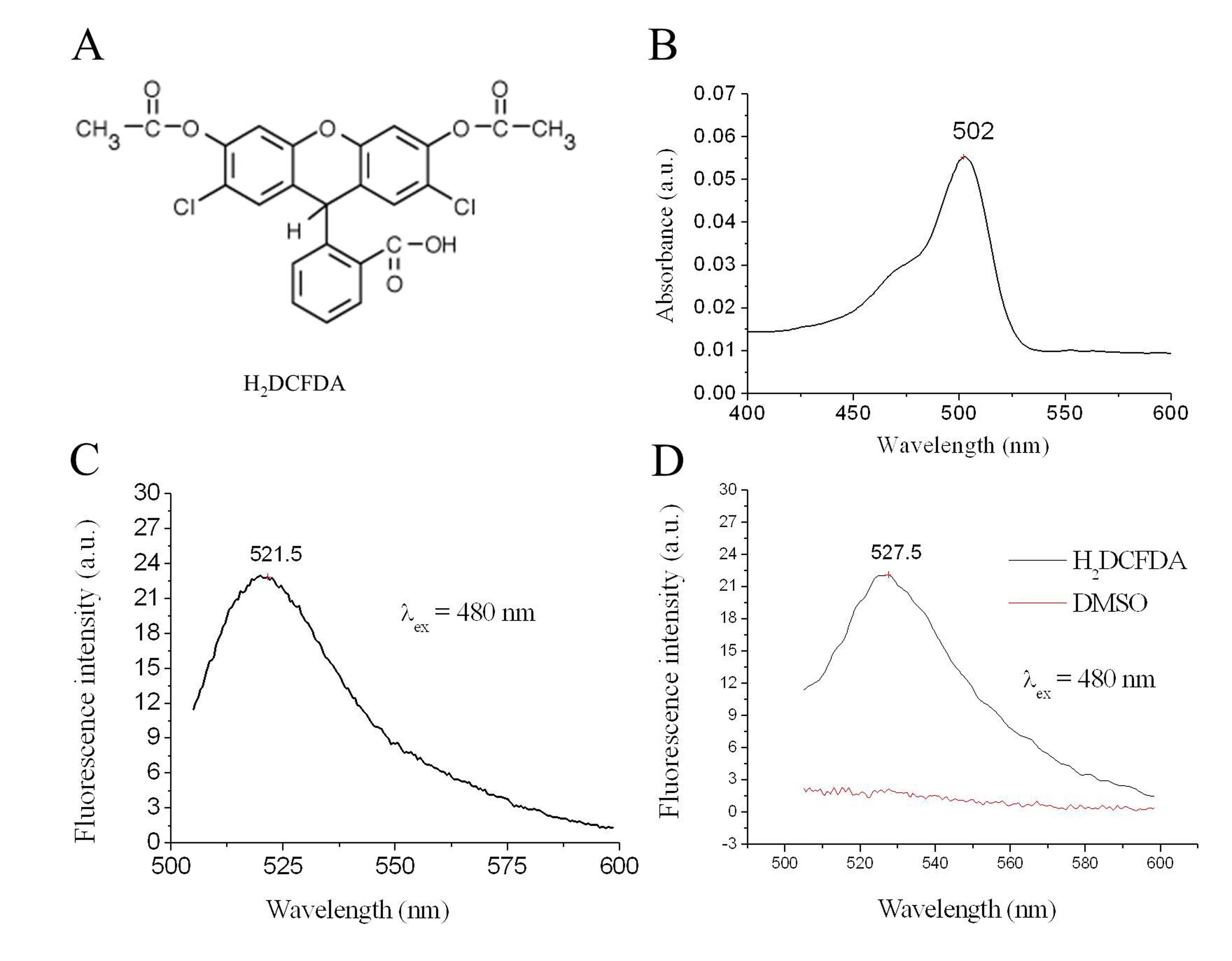

Supplement: Supplementary Figure 1 — Structure and spectral characteristics of H2DCFDA. (A) Molecular structure of H2DCFDA. (B) The absorption spectrum of 10 μ M H2DCFDA in 0.2% DMSO solution shows a maximum absorption peak at 502 nm. In all experiments, however, we used 480 nm as the excitation light to avoid the effect of the excitation light on the fluorescence emission. (C) Fluorescence emission spectra of 10 μ M H2DCFDA in 0.2% DMSO solution with excitation at 480 nm. (D) Fluorescence emission spectra of A. thaliana leave after incubation with 10 μ M H2DCFDA in 0.2% DMSO solution. The samples were excited at 480 nm, and the spectra show a 6 nm redshift when compared with (C). All spectra were obtained using an LS55 spectrophotometer in the presence of 10 μ M H2O2 applied exogenously to oxidize the H2DCFDA. [file Image1.JPEG]

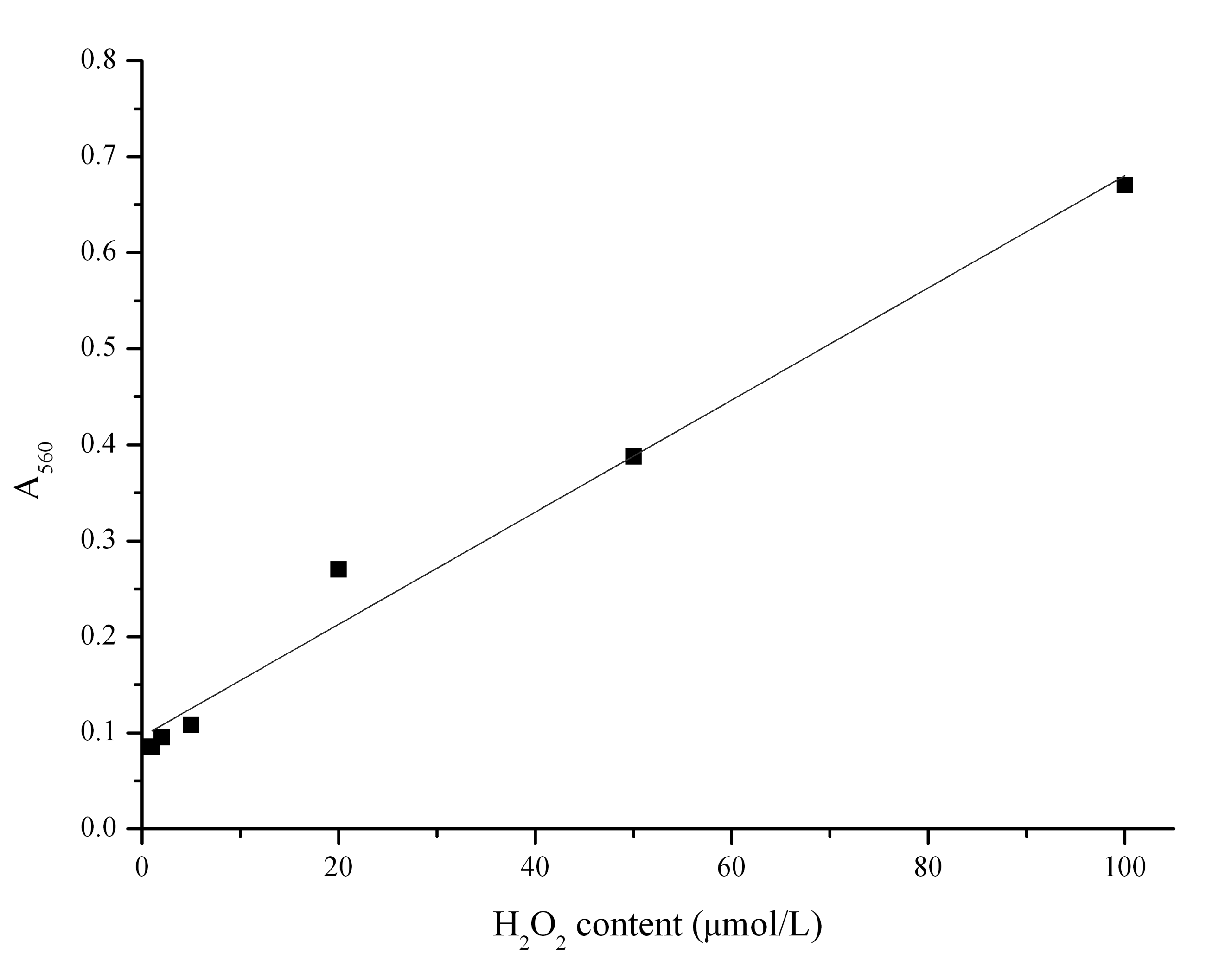

Supplement: Supplementary Figure 2 — Standard concentration curve of H2O2. [file Image2.TIF]
